# Leaders’ Role in Shaping Followers’ Well-Being: Crossover in a Sample of Nurses

**DOI:** 10.3390/ijerph20032386

**Published:** 2023-01-29

**Authors:** Andrea Caputo, Paola Gatti, Marco Clari, Giacomo Garzaro, Valerio Dimonte, Claudio Giovanni Cortese

**Affiliations:** 1 Department of Psychology, University of Turin, 10124 Turin, Italy; 2 Department of Psychology, University of Milano-Bicocca, 20126 Milan, Italy; 3 Department of Public Health and Pediatrics, University of Turin, 10124 Turin, Italy

**Keywords:** nurse managers, nurses, work engagement, job satisfaction, crossover, transformational leadership, conservation of resources, multilevel mediation

## Abstract

The link between well-being at work and leadership has received considerable attention. Leaders have the power to influence followers not only due to formal position, but also their positive behaviors could reinforce the followers’ positive working experience. Following the crossover model (Westman, 2001), this study investigates whether leaders’ work-related positive psychological states (i.e., work engagement) cross over to those of the followers (i.e., work engagement and job satisfaction) through the mediation of the latter’s perception of transformational leadership. We used MPlus 8 to test two multilevel mediations in a sample of 1505 nurses nested in 143 groups led by as many leaders (87.19% of nurses and 56.50% of head nurses of the entire population). Results show that while there is not a crossover of leader work engagement to nurse work engagement, manager work engagement can cross over to nurse job satisfaction, enhancing their well-being through transformational leadership behaviors. This study adds further insights both on crossover theory and on the importance of leaders in expanding and transferring resources to followers at work. Fostering work engagement at a managerial level in the healthcare sector could be the driver to facilitate the well-being of nurses at work, address negative outcomes, and promote positive ones.

## 1. Introduction

Nurses generally face numerous high demands at work that can be detrimental to their general health and well-being at work [1,2]. Were this not enough, the COVID-19 pandemic meant that nurses have had to work under “extreme and persistent psychological pressure since they are particularly exposed to the threat of SARS-CoV-2 infection, and they have become overwhelmed by fear for the safety of their own health, their close family members, and their patients [3]” [4] (p. 2). Given this pressure—and nurses’ crucial role in the response to the pandemic—investigating their well-being is more important than ever.

Leaders play a fundamental role for nurses [5]: constructive and relationship-focused leadership has been shown to have positive effects on nurses’ attitudes and behaviors, patient satisfaction, and quality of care [6,7]. Cummings and colleagues’ [8] review of the nursing leadership literature found that leadership styles focusing on people and relationships (e.g., transformational, supportive, resonant, and consideration styles) have a positive connection with higher nurse job satisfaction, enhanced staff well-being, and greater organizational productivity and commitment. Accordingly, “from the viewpoint of nursing management, nurse leaders have a key role in promoting well-being at work” [9] (p. 738), and leaders in different work contexts have a vital role in combating COVID-19 [10]. It is, thus, likely that the need for leadership is particularly high in the healthcare sector and merits further investigation.

While the leadership literature generally holds that leaders’ behavior influences followers’ health and well-being [11], scholars have devoted little attention to a specific mechanism linking leadership and the well-being outcomes of followers, viz., crossover of positive psychological states from leaders to followers in their work team [12]. Furthermore, drawing on the conservation of resource theory (COR theory; [13,14]), our study aims to contribute to the literature investigating the positive side of this theory, i.e., the gain spiral. By experiencing a gain spiral, where an individual experiences a resource gain [14], engaged leaders could be capable of establishing good relationships with their followers [12] and engage in supportive behaviors [15]. In this way, followers could perceive the positive behaviors of their leader as a resource, thus enhancing their well-being.

This study tests the indirect crossover model described by Westman [16], which hinges on the influence of certain mediators. Specifically, we will focus on the positive crossover from the well-being of leaders (i.e., work engagement) to followers (measured as both work engagement and job satisfaction) through the mediation of transformational leadership. This active–constructive leadership style [17] could explain how motivated leaders engage in positive leadership behaviors with followers and transfer their well-being to them.

Many studies focusing on the nursing profession have shown a link between workers’ well-being (i.e., work engagement and job satisfaction) and outcomes of considerable interest linked to the staff’s physical health, to managerial and organizational issues, and to the quality of the service provided. From the point of view of work engagement, workplace literature shows how this motivational state is linked to important outcomes, such as job performance [18], as highlighted in the job demands–resources theory [19], financial benefits [20], and commitment and health [21]. In the nursing literature, the review by Keyko and colleagues [22] states that in a context where there is continual pressure to reduce errors and to improve patients’ quality of care, nurses’ work engagement is essential for ethical nursing practices [23] and to reinforce both nurse and nurse manager behaviors to create a safe environment for patient care [24]. From the point of view of worker job satisfaction, outcomes include burnout [25] and work stress [26]. Retention [27], turnover intentions [28], and job performance [29] are examples of organizational and managerial outcomes. Lastly, there is a proven relationship between nurse job satisfaction and patient satisfaction with the service offered [7], which, in turn, can be considered an indicator of quality of service. All these outcomes of nurse work engagement and job satisfaction make the value of studying these constructs very clear, as highlighted particularly in these difficult times [30].

To sum up, drawing on crossover [16] and COR theory [13,14], this study investigates a mediation that explains the well-being of nurses through the crossover triggered by leaders’ work engagement, mediated by transformational leadership. The study, thus, sheds light on a process that can lead to the well-being of nurses through the influence of “engaged leadership”, both of which call for particular attention in this time of crisis.

## 2. The Leader’s Work Engagement as Crossover “Driver”

### 2.1. Crossover Model and Conservation of Resources Theory

In view of the importance of well-being for nurses, our study focuses on the active role of engaged leaders who can transfer their positive psychological states to their followers by enacting a positive leadership style and being perceived as such by followers. The crossover model [16] can provide an explanation of the process underlying the transmission of these positive psychological states between people sharing the same social environment of Refs. [14,31]. The model describes an inter-individual level mechanism of transmission, acting in dyads, teams, or at the organizational level, whereby experiences, emotions, resources, and psychological states are transferred to other individuals within the same social context [32]. Crossover was originally defined as the interpersonal process that occurs when job stress or psychological strain experienced by one person affects the level of strain of another person in the same social environment [31]. In line with this first definition, the initial studies on crossover focused on variables, such as anxiety [33], depression [34], and burnout [35]. These studies investigated crossover of negative emotional and psychological states, while few efforts were made to analyze the transfer of positive affects and positive experiences [32]. Nevertheless, in her foundational contribution, Westman [16] suggested broadening the definition of crossover, hypothesizing that even positive experiences could be transferred. In addition, she wrote an editorial on positive crossover [36]. Thus, this investigation aims to enrich the limited amount of literature on the transfer of positive states from leaders to followers [12].

While crossover was initially investigated in the context of family relationships [31,37]), the construct was soon transferred to the workplace [15,38]. In this context, however, little attention was given to crossover in leadership relationships. There have been a few studies on crossover in leader–follower dyads [39], a small number using a multilevel perspective to investigate crossover from leaders to their group of followers [12,40,41,42], and a handful focusing on crossover from followers to leaders [43]. Nevertheless, it is well-known that leaders “are in a unique position to influence subordinates’ emotions” [44] (p. 1058) because the nature of the leader role itself is substantiated in the social interaction with followers [43,45]. Leaders can strongly influence followers’ attitudes, affects, and behaviors: for example, their formal authority enables them to make important decisions influencing followers’ life at work [41]; in addition, they can be an example to follow or a role model [12] for their followers, thus exerting a broad spectrum of influence. Furthermore, leader and followers are parts of the same social system [43] and, as Bronfenbrenner’s [46] system theory suggests, the “components within the system tend to interrelate and affect each other” [16] (p. 719). In the leadership literature, crossover has been investigated in the transfer of variables of strain, such as burnout [39,40] and psychological distress [41], while, to the best of our knowledge, the only study to date that applies the crossover model and investigates a positive transfer in leadership is that by Gutermann and colleagues [12], which focuses on the crossover of work engagement from the leader to followers.

In view of the weight of evidence demonstrating that emotional, affective, and psychological states can cross over between leaders and followers, it is important to investigate how and under what conditions this transfer takes place. Westman’s [16] model suggested three processes of crossover: a direct process through empathy “between closely related partners or team members who care for each other and share the greater part of their lives together” [32] (p. 98); an indirect process through mediators and moderators; and a spurious process due to common stressors shared by the people involved in the process and impacting on both individuals’ affective states. Studies on crossover in the leader–follower relationship have focused on the role of mediating variables in the indirect crossover process. Westman [16] proposed three clusters of mediators, viz., personal attributes (e.g., gender, life stages, and workaholism), interpersonal factors (e.g., coping strategies, social support, social undermining, and communication), and common stressors (e.g., life events). Studies on leadership have shown how crossover of leader’s emotions and negative and positive states on followers’ negative and positive emotions could be mediated by followers’ negative/positive affect [40], job and personal resources [39], or perceptions about their leaders’ leadership style, such as abusive supervision [41], supportive behaviors [40], or about the quality of interaction with their leader, using LMX [12].

While crossover theory can explain how there can be a transference of psychological states from one individual to another (or from one to many), the Conservation of Resources theory (COR) [13,14] could explain why. Indeed, in the literature, these two theories are usually placed side by side [14,41]. COR theory is a motivational theory that “begins with the tenet that individuals strive to obtain, retain, foster, and protect those things they centrally value” [14] (p. 104), i.e., their resources. Resources are defined as personal characteristics, objects, energies, and all features that people value as important to themselves [41,47]. Usually investigated on the negative side, COR theory posits that when individuals experience a resource loss, they will experience many negative consequences, for instance, anxiety, burnout [39], reduced job performance, and decreased job satisfaction [41]. Thus, when experiencing resource depletion, people commit to maintaining and protecting remaining resources. Hobfoll [47] describes this process as a “loss spiral”, because “at each stage of the stress process people are increasingly vulnerable to negative stress sequelae” (pp. 337–338), thus resulting in a spiral.

### 2.2. Gain Spirals and Work Engagement

Whereas resource loss is more salient and impactful, the opposite side is a “gain spiral”: individuals who experience an increase of resources due to positive job characteristics have a greater chance of benefiting from those resources [14,48]. Thus, whereas people in loss spirals feel more vulnerable and, in order to avoid future resource loss, tend to exhibit negative behaviors (e.g., abusive supervision [41]), people in gain spirals could feel more engaged, being more capable of performing positive behaviors [14], e.g., transformational leadership.

The psychological state which can represent a resource gain and which can trigger a positive crossover is work engagement [14]. In workplace literature, work engagement is defined as “a positive, fulfilling, work-related state of mind that is characterized by vigor, dedication, and absorption” [49] (p. 74). Engagement literature shows how work engagement is predicted by job resources (e.g., autonomy, independence at work, and social and supervisor support) and personal resources (e.g., self-efficacy; cf. job demands–resources theory; [19]). Furthermore, crossover literature shows how engaged individuals are capable of expressing their engagement with their partners/colleagues [14], performing a positive crossover of engagement (e.g., [15]). In addition, we hypothesize that engaged leaders can more easily express constructive leadership behaviors (i.e., transformational leadership; [17]).

## 3. The Crossover Process and the Mediating Role of Leadership Style

Holding the formal position of leader is not a sufficient premise to express positive leadership behaviors [17]. In order to be a transformational leader, it is necessary, on the leader’s side, both to be motivated, “being engaged with work and of solving problems with enthusiasm and open mindedness” [50] (p. 31), and to be perceived as such by followers. Gilbert and Kelloway [17] tested the validity of this assumption and showed the relationships between leaders’ motivation and followers’ perceptions of active–constructive leadership behaviors, such as transformational leadership.

The role of motivation as an antecedent of transformational leadership has already been hypothesized in some studies in the literature [51]. However, more clarification is needed regarding all the characteristics of the leader and the context of work that can be considered as antecedents of effective transformational leadership [52]. Therefore, in the present study, the link between leader’s work engagement (see Job Demand–Resource theory, [19]) as an antecedent of followers’ perceptions of the leader’s leadership style was tested.

Moreover, it has only recently been found that leaders can transfer their distress to followers by displaying behaviors perceived as destructive [41]. We hypothesize that the opposite process can also take place: a positive motivational state at work (i.e., work engagement) experienced by leaders could lead them to display positive behaviors (specifically, transformational leadership) with their followers, making the latter more engaged in and more satisfied with their work, thus influencing their well-being. Engaged leaders “may be great at expressing enthusiasm, radiating positive emotions and inspiring others” [49] (p. 941), consequently showing positive leadership behaviors, perceived as such by followers, and transferring positive feelings to them at work. “Inspiring” followers through one’s motivation is one of the four components of transformational leadership: inspirational motivation, together with idealized influence, intellectual stimulation, and individualized consideration, concurs in building this leadership style [53,54].

On the other hand, the importance of transformational leadership on employees’ well-being has been widely investigated in the literature. Transformational leaders can contribute to employees’ engagement, that is, to enhance their intrinsic motivation to perform at work because they enjoy it [55]. For instance, transformational leaders provide followers with meaning for their work and stimulates them to prioritize group interests over personal ones [56]. Employees dealing with transformational leaders who give support, inspiration, and coaching are likely to “experience work as more challenging, involving and satisfying, and consequently, to become highly engaged” [57] (p. 123). This is also true for nurses: transformational leaders can enhance nurses’ engagement in the workplace, e.g., via fostering their self-efficacy [58] or their quality of work life [59].

As regards the link between transformational leadership and job satisfaction, it has likewise been widely studied (for a review of this specific relationship among hospital staff, see Hussain and Khayat, 2021 [60]). Multilevel studies have shown that perceiving a leader as transformational can have positive effects on both individual and group job satisfaction [61,62]. Transformational leadership, when investigated on individual followers, shows its effectiveness on different outcomes, e.g., psychological empowerment [63], burnout [64], and, of course, job satisfaction [62,65], even when complex models presenting other predictors are used [66]. When measured at the group level, transformational leadership has effects on outcomes, such as group performance [63], exhaustion [67], and, again, on job satisfaction [62,68]. Focusing on the nursing literature, the importance of the job satisfaction of nurses can be discovered referring to some of its more practical outcomes and topics of interest of nursing human resource management. Recent studies have shown, for example, that transformational leadership can enhance, via affecting job satisfaction, nurses’ willingness to stay [69] and the quality of care [7].

## 4. Crossover of Well-Being at Work, from Leaders to Followers

In light of these considerations, it seems possible to hypothesize that a crossover of well-being from leaders to followers could occur, triggered by an engaged leader.

On the one hand, engaged leaders could express their engagement in interactions with their followers [14] by establishing good relationships with them [12] or expressing positive, active–constructive behaviors [17], thus transferring engagement and its components. On the other hand, this study focuses on the crossover of positive psychological states at work, investigating two different variables linked to well-being at work in the followers’ sample: work engagement and job satisfaction.

Though conceptually different, both work engagement and job satisfaction are positive indicators of subjective work well-being [70,71]. They are the two positive states which make up the positive half of the circumplex model of affect [70], characterized by a different degree of activation. While work engagement shows “high levels of pleasure and activation” (p. 6), job satisfaction “reflects a high level of pleasure and a low level of activation” (p. 9). Previous studies have shown that a leader is better able to transfer these positive feelings to followers when displaying high-arousal positive affect (such as enthusiasm, which might fall within the engagement segment of the circle) rather than low-arousal positive affect (such as relaxation, which might fall within the satisfaction segment [72]).

Moreover, work engagement and job satisfaction are both included as measures of work-related well-being also according to Warr’s [71] model, suggesting that well-being can be examined along three dimensions: pleasure–displeasure (where job satisfaction can be found), anxiety–comfort, enthusiasm–depression, and a possible fourth dimension named fatigue–vigor (where work engagement can be found [73,74]).

Considering that “studies distinguishing high and low arousal affective states are especially scarce” [72] (p. 2607), that crossover between leaders’ work engagement and followers’ work engagement has been demonstrated [12], and that job satisfaction is of considerable importance in the nursing context [7] as well as work engagement [22], this study aims to add to the literature findings regarding the crossover of leader well-being to follower well-being, described in both their work engagement and job satisfaction.

In light of the discussed literature, we tested two multilevel mediation models, where the antecedent and the mediator are the same (i.e., leaders’ work engagement and transformational leadership, respectively), while the outcome changes. Model 1 tests the crossover of well-being from leader work engagement to follower work engagement. Below, we summarize the hypotheses for Model 1:

**Hypothesis 1a** **(H1a).** *Leaders’ work engagement is positively related to follower’s perceptions of transformational leadership*.

**Hypothesis 1b** **(H1b).** *Follower’s perceptions of leaders’ transformational leadership are positively related to followers’ work engagement*.

**Hypothesis 1c** **(H1c).** *The positive relationship between leaders’ work engagement and followers’ work engagement is mediated by follower’s perceptions of transformational leadership*.

Model 2 tests the crossover of well-being from leaders’ work engagement to followers’ job satisfaction. Below, we summarize the hypotheses for Model 2:

**Hypothesis 2a** **(H2a).** *Leaders’ work engagement is positively related to follower’s perceptions of transformational leadership*.

**Hypothesis 2b** **(H2b).** *Follower’s perceptions of leaders’ transformational leadership are positively related to followers’ job satisfaction*.

**Hypothesis 2c** **(H2c).** *The positive relationship between leaders’ work engagement and followers’ job satisfaction is mediated by follower’s perceptions of transformational leadership*.

## 5. Materials and Methods

### 5.1. Procedure and Participants

Paper and pencil questionnaires were administered to both nurses and nurse managers working in hospitals of northwestern Italy. The questionnaire was developed for a broader project titled “Sentirsi Leader” (i.e., “Feeling like a leader”), which investigated leadership relationships also considering other features of leaders (e.g., leader identity). In order to match and combine the data of nurse managers with their followers’ groups, alphanumeric codes were generated by the researchers. All participants were informed of the procedure by invitation letters and information sheets attached to each questionnaire. Before beginning the data collection, the project received approval firstly from the director of the Directorate of Health Professions and the nursing managers of the target organization (allowing the involvement of both head nurses and their followers), and secondly by the Bio-Ethics Committee of the University of Turin (Approval letter, Prot. No. 55631 of 1 February 2019). Head nurses were invited by receiving an e-mail with the information sheet introducing the research; after agreeing to participate, two administrators went on-site to deliver paper copies of the questionnaires in person to the head nurse, which were immediately collected once completed. Then, nurses completed their version of the questionnaire, signed with an alphanumeric code, and placed it in a blank envelope; all blank envelopes from each ward were collected and retrieved by the administrators themselves. Administration and data collection were held from February to May 2019.

According to the procedure, the objective of the project was to involve the entire population of nurses and head nurses of the target hospitals. The total population counted 164 head nurses and 2664 nurses. Inclusion criteria for the sample of this study were: head nurses agreeing to fill in their questionnaires; at least three nurses per group filling in the questionnaire; and at least the 61% of all items being clearly completed. According to these criteria, the total sample of this study consisted of 143 nurse managers and 1505 nurses working in four hospitals, with a response rate (related to the entire population) of 87.19% for head nurses and 56.50% for nurses. As regards the sample of nurse managers, 83.9% were women and 16.1% men. As regards educational level, 76.9% of the sample had a professional nursing school diploma, 7.7% a bachelor’s degree, and 15.4% a master’s degree, while 69.2% attended a one-year course on nursing management. The sample average age was 53.07 years (SD = 5.32), with an average length of employment of 32.39 years (SD = 6.26) and an average organizational tenure of 29.67 years (SD = 8.58). The average tenure in the leadership role is 12.74 years (SD = 8.43), ranging from a minimum of less than 1 year to a maximum of 38 years. Table 1 summarizes the characteristics of the sample.

As regards the sample of 1505 nurses, 82.5% were women and 17.5% men, and they worked in 143 groups, each group led by one nurse manager. The average size of the groups was 19.27 participants (with SD = 10.62), ranging from a minimum of 3 to a maximum of 70 nurses per group. As regards educational level, 52.6% of the nurses had a professional nursing school diploma, 42.7% a bachelor’s degree, and 4.7% a master’s degree. The sample average age was 43.41 years (SD = 9.17), with an average length of employment of 20.6 years (SD = 9.85) and an average organizational tenure of 16.94 years (SD = 10.24). Additionally, considering nurses shifts, 21.9% of the sample worked on two shifts, 56.3% on three shifts, while 21.8% did not work shifts. Table 1 summarizes the descriptive results.

### 5.2. Measures

Work engagement (measured on leaders and followers) was measured with the short version of the Utrecht Work Engagement Scale (UWES [75]), consisting of nine items, using a 7-point Likert scale (from 0 = “never” to 6 = “always”). This scale assesses the three subcomponents of work engagement (vigor, dedication, and absorption). An example item for each subdimension is: “At my work, I am bursting with energy” (vigor); “I am enthusiastic about my job” (dedication); “I am immersed in my work” (absorption). Cronbach’s α for the leaders’ sample was 0.92, with explained variance of factorial solution (estimated with Maximum Likelihood method) of 58.30%, while for the followers’ sample, it was 0.90 (explained variance of exploratory factor solution = 53.49%).

Transformational leadership (followers) was assessed with the 7-item scale by Carless and colleagues [76], using a 7-point Likert scale (from 1 = “strongly disagree” to 7 = “strongly agree”). An example item is “(Your nurse manager …) treats staff as individuals, supports and encourages their development”. Cronbach’s α = 0.97 (explained variance of factorial solution = 82.69%).

Job satisfaction (followers) was measured by the scale proposed in the Copenhagen Psychosocial Questionnaire (COPSOQ [77]), consisting of 4 items, using a 5-point Likert scale (from 1 = “very dissatisfied” to 5 “very satisfied”). Respondents were asked, for example, “(how satisfied are you with …) the way your abilities are used?” One item assessing the satisfaction of respondents with their working relationships was added to this scale by the researchers. Cronbach’s α = 0.87 (explained variance of factorial solution = 58.23%).

State positive affectivity (followers) was assessed as control variable using the 10-item scale by Terracciano and colleagues [78], the Italian translation of the original scale by Watson and colleagues [79]. Respondents were asked to indicate the extent to which they felt, for example, “Interested” or “Enthusiastic” on the day when they were filling in the questionnaire. Cronbach’s α = 0.89 (explained variance of factorial solution = 45.46%).

### 5.3. Data Analysis

SPSS 28 (IBM, Armonk, NY, USA) and MPlus 8 (Muthén & Muthén, Los Angeles, CA, USA) were used to perform data analyses. SPSS was employed for descriptive analyses of the sample and of the study variables, internal consistency with Cronbach’s α, ANOVA, and exploratory factor analysis (for both psychometric properties of the instruments and the common method bias investigation). MPlus 8 was used to test the hypothesized multilevel random intercept model, with head nurses’ WE as Level 2 (leader/group level) predictor, while Level 1 (individual level) variables (TL, JS, and nurses’ WE) were decomposed into their latent variables at the between and within levels, in order to calculate also indirect effects [80].

## 6. Results

Means and standard deviations of study variables are presented in Table 2, which also shows intraclass correlation coefficients (ICC(1)) and correlations between variables at the within level. In this regard, the dummy variable of pediatric ward affiliation was used as a control variable at the between level. The ANOVA performed with Bonferroni correction for post hoc comparisons showed that those working in a pediatric ward had significantly higher mean scores on work engagement (F = 10.29, *p* < 0.001) than those working in Medicine (*p* < 0.001), Surgery (*p* < 0.05), and E.R. (*p* < 0.001); similarly, pediatric nurses showed higher mean scores on job satisfaction (F = 3.91, *p* < 0.01) than nurses working in Medicine (*p* < 0.05).

ICC(1) for the outcomes was calculated, which measured the percentage of variance of the lower level dependent variable [81]. The ICC(1) for followers’ work engagement was very low (ICC(1) = 0.057); thus, the authors chose not to proceed with the multilevel analyses since only the 5.7% of the variance for work engagement was attributable to the division into groups. Considering these findings, and specifically due to the very low intraclass correlation coefficient (ICC(1)) of the followers’ work engagement, we chose to present only Model 2 and to move Model 1 to the Appendix A. On the other hand, the ICC(1) of the followers’ job satisfaction was 0.084, meaning that the 8.4% of the variance resided between groups [82].

**Figure 1:**
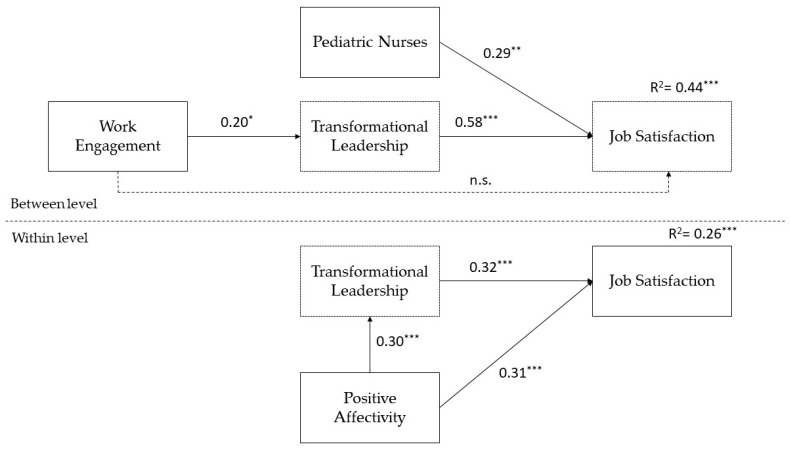
Results of Model 2 with standardized estimates. Note: * *p* < 0.05; ** *p* < 0.01; *** *p* < 0.001. Discontinuous lines indicate non-significant relationships.

Figure 1 shows the results of the analyses on the multilevel Model 2.

The model showed a good fit: χ^2^_(2)_ = 4.461, *p* = 0.11; Comparative Fit Index (CFI) = 0.99; Tucker–Lewis Index (TLI) = 0.98; Root Mean Square Error of Approximation (RMSEA) = 0.03; and Standardized Root Mean Square Residual (SRMR) for between level = 0.07 and for within level = 0.00. As regards these indices, CFI and TLI > 0.90 indicated a good fit of the model, as did values of RMSEA < 0.08 and of SRMR < 0.08 [83].

Results highlighted that, at the between level, leaders’ WE had a positive relationship with followers’ perceptions of TL (*β* = 0.20, *p* < 0.05), confirming H2a; TL was positively related to the followers’ JS (R^2^ = 0.44, *p* < 0.001; *β* = 0.58, *p* < 0.001), also controlling for the nurses’ affiliation to the pediatric wards (*β* = 0.29, *p* < 0.01), as previous studies reported that pediatric nurses could experience higher levels of JS than other nurses [84], so H2b has been confirmed. There was not a significant direct relationship between WE and JS, while there was an indirect positive relationship (*β* = 0.12, *p* < 0.05), confirming hypothesis H2c. At the within level, TL (R^2^ = 0.08, *p* < 0.001) was positively related to JS (R^2^ = 0.26, *p* < 0.001; *β* = 0.32, *p* < 0.001), also controlling for PA both on TL (*β* = 0.30, *p* < 0.001) and on JS (*β* = 0.31, *p* < 0.001); and there was an indirect effect of PA on JS through TL (*β* = 0.09, *p* < 0.001).

## 7. Discussion

This study contributes to the literature regarding crossover in leadership relationships. Following the conservation of resources [13,14] and the crossover theory [16], this study provides novel insights into the positive influence that engaged leaders have on followers’ well-being. In fact, results show that an engaged leader is able to enact a positive leadership style such as transformational leadership and, thus, to be perceived as such by followers. Moreover, this leadership style is positively related to the nurses’ well-being, as highlighted in the previous literature [57,58,59].

### 7.1. Theoretical Implications

From a theoretical standpoint, the main finding of this work is that the well-being of an engaged leader can cross over to followers, fostering their job satisfaction by enacting a positive leadership style, even when the model considers control variables which are linked to well-being at work and are able to tap an individual dimension (PA) and a contextual dimension (the clinical area in which the nurses work). This finding enriches the literature on positive crossover, as studies on this mechanism applied to leadership relationships focused mainly on the negative side [40,41].

Furthermore, this study adds a contribution to recent findings regarding the positive crossover of well-being [12], which has been divided into its two components of motivation and satisfaction, as suggested by the circumplex model [70,74]. In line with previous studies highlighting that work engagement can be the drive of crossover [12], our study showed that engaged leaders are able to transport their well-being to followers via transformational leadership. In other words, inspired by a gain spiral [14], engaged leaders can trigger the crossover of their positive psychological states to followers via their transformational leadership behaviors, e.g., inspiring and encouraging followers to “go beyond their self-interests or expected reward for the good of the team and the good of the organization” [54] (p. 118).

### 7.2. Practical Implications

Our findings suggest that promoting a culture of engagement, starting from head nurses, could be fruitful in affecting nurses’ wellbeing. From the healthcare organizations’ point of view, training paths for head nurses could be implemented to enhance their engagement, together with regular (perhaps annual) surveys to monitor this aspect. Head nurses’ wellbeing contributes to a healthy workplace, and their empowerment could help them to express more effective transformational leadership behaviors, which, in turn, promote followers’ wellbeing, with subsequent implications on better quality of care provided and nurse retention [7].

### 7.3. Limitations

This study is not exempt from some limitations. First, this study has a cross-sectional design, which does not allow for inference of the causal effects among study variables [85,86]. Second, relying on self-reported data implies the chance of incurring the issue of common method bias. To alleviate this concern, on the one hand, data were collected from multiple sources (i.e., leaders and their direct followers); on the other hand, according to the Harman’s single-factor test [87], an exploratory factor analysis (principal component method of extraction; [88]) including all the measures of our study bore out two factors, accounting for 56.25% of the total variance, with the first factor accounting for 38.18% and the second factor for 18.07%. Therefore, there is not a single factor accounting for the majority of the variance, and these two reasons suggest that common method bias was not an issue for this study [87]. Third, the short form of the transformational leadership scale does not allow for the investigation of the sub-dimensions of this construct that could have clarified further the mediation and its functioning. Fourth, as anticipated, the low ICC(1) of the followers’ work engagement led us not to proceed with the interpretation of multilevel analysis of Model 1 (presented in the Appendix). Finally, although presenting results before the COVID-19 crisis, this study highlights the importance of the head nurses’ role in shaping followers’ well-being. Post-crisis studies have shown how the importance of this leaders’ role within their relationship with followers (which declined in the enactment of different leadership styles) was even reinforced by the consequences that the pandemic had on the health care system [89,90,91].

### 7.4. Future Research

Future studies could explore the functioning of positive crossover in greater detail by considering “caravan passageways of resources” from leaders to followers, as highlighted by the conservation of resource theory and triggered by the gain spiral [14], also controlling for other important variables for nurses, for example, individual characteristics or working on shifts. Furthermore, positive crossover could be investigated also in leader–follower dyadic relationships.

## 8. Conclusions

In summary, this study shows that a positive crossover [16,36] of well-being from leaders to followers can occur. Going beyond previous studies which focused mainly on the negative aspect of crossover [40,41], leaders can be both source and facilitators of followers’ positive psychological states at work. Engaged leaders foster a work context where employees thrive [92], so it is important to study the ability of nurse leaders to affect nurses’ well-being due to their key role. The role of nurse leaders is fundamental since they are one of the main sources of nurses’ engagement [93] and satisfaction [94], acting also as a protection against nurse exhaustion [95]; consequently, nurses could provide good patient quality of care [96]. From a practical point of view, these considerations suggest that health care management should focus on improving training activities for nurse leaders regarding leadership topics and on having engaged nurse leaders who feel that they master the necessary resources to cope with everyday work.

In light of these considerations, leaders should be aware that they play a key role in which their psychological states could affect those of nurses. This is crucial, considering that nurses’ psychological well-being is the prelude of effective performance, from which the whole health care system benefits [97]. It is, thus, vital for health care management to empower the sources that can improve nurses’ well-being, which has deteriorated during the recent fight against COVID-19 [98].

## Figures and Tables

**Table 1:** Descriptive results of the sample.

Socio-Demographic Variables		Leader Sample (N = 143)				Follower Sample (N = 1505)			
		N	%	M	SD	N	%	M	SD
Gender	F	120	83.9			1229	82.5		
	M	23	16.1			261	17.5		
Education level	Professional nursing school diploma	110	76.9			781	52.6		
	Bachelor’s degree	11	7.7			633	42.7		
	Master’s degree	22	15.4			70	4.7		
Age				53.07	5.32			43.41	9.17
Length of employment				32.39	6.26			20.6	9.85
Organizational tenure				29.67	8.58			16.94	10.24
Shifts	No shifts					326	21.8		
	1 shift					328	21.9		
	2 shifts					841	56.3		
Clinical area	General Medicine	54	37.8			532	35.3		
	Surgery	51	35.7			448	29.8		
	E.R.	12	8.4			225	15.0		
	Pediatrics	26	18.2			300	19.9		
Tenure in leadership role				12.74	8.43				

Note: All means refer to years.

**Table 2:** Correlations of variables.

		Variable	M	SD	ICC	1.	2.	3.	4.	5.	6.
Followers	1.	JS	3.33	0.79	0.084	-					
	2.	WE	3.92	1.09	0.057	0.58 ***	-				
	3.	TL	4.47	1.66	0.283	0.42 ***	0.30 ***	-			
	4.	PA	3.56	0.72	-	0.40 ***	0.51 ***	0.27 ***	-		
Leaders	5.	WE	4.10	1.00	-	0.07 ***	0.05 *	0.13 ***	0.06 *	-	
	6.	Pediatrics^1^	-	-	-	0.06 *	0.12 ***	−0.07 **	0.01	−0.03	-

* *p* < 0.05; ** *p* < 0.01; *** *p* < 0.001; 1 Pediatrics = 1, other wards = 0.

**Figure A1:**
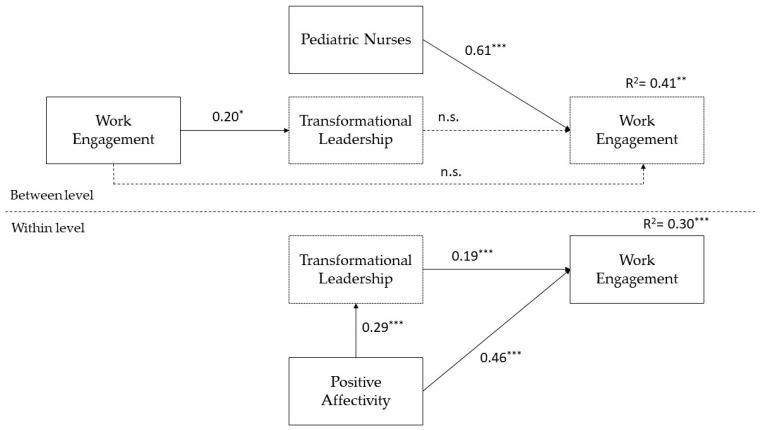
Results of Model 1 with standardized estimates. Note: * *p* < 0.05; ** *p* < 0.01; *** *p* < 0.001. Discontinuous lines indicate non-significant relationships.

## Data Availability

Data sharing is not applicable to this article, to avoid any potential recognition of head nurses and groups.

## Appendix A

The low ICC(1) of nurses’ work engagement (0.057) led us not to proceed with multilevel analyses [82]. For the sake of completeness, we show the results of Model 1 in Figure A1. This model shows good fit indices (χ^2^_(2)_ = 4.345, *p* = 0.12; CFI = 0.99; TLI = 0.98; RMSEA = 0.03; and SRMR for within level = 0.00 and for between level = 0.07); however, the low ICC(1) of the dependent variable still led us not to comment on the results. In addition, the direct and indirect relationships of leaders’ WE on followers’ WE are both non-significant.
